# Efficacy and safety of the OZil phacoemulsification handpiece in dropped lens fragment surgery: a randomized controlled trial

**DOI:** 10.22336/rjo.2024.52

**Published:** 2024

**Authors:** Vipin Rana, Deependra Kumar Sinha, Meenu Dangi, Atul Gupta, Sandepan Bandopadhyay, Jaya Kaushik, Ashok Kumar, Amit Nandan Tripathi

**Affiliations:** 1 Department of Ophthalmology, Command Hospital (Eastern Command), Kolkata, India,; 2 Department of Anaesthesia, Command Hospital (Eastern Command), Kolkata, India,; 3 Department of Ophthalmology, Command Hospital (Central Command), Lucknow, India,; 4 Western Eye Hospital, London, United Kingdom,; 5 Department of Ophthalmology, Armed Forces Medical College, Pune, Maharashtra, India

**Keywords:** OZil, phacoemulsification, nucleus drop, cataract, phacofragmentation, BCVA = Best corrected visual acuity, CME = Cystoid macular edema, PPV = Pars plana vitrectomy, PFCLs = Perfluorocarbon liquids

## Abstract

**Objective:**

Phacoemulsification is the predominant method for cataract surgery, but complications like lens nuclei dislodgment into the vitreous cavity pose significant risks, including inflammation, glaucoma, retinal tears, and vision loss. Traditional management involves pars plana vitrectomy with phacofragmentation, which can increase the risk of retinal damage due to repulsive forces. This study tests whether the OZil phacoemulsification handpiece, employing torsional movement, offers a safer alternative by minimizing repulsive forces and reducing surgical duration compared to the traditional phacofragmatome.

**Methods:**

This prospective study, conducted in a tertiary care hospital in eastern India from January to June 2023, enrolled 40 patients with nucleus dislocation during cataract surgery. Patients were randomized into Group 1 (traditional phacofragmatome) and Group 2 (OZil handpiece). Primary objectives included comparing the duration of surgery and intraoperative complications. Secondary objectives assessed postoperative best-corrected visual acuity (BCVA), surgical site safety, and cystoid macular edema (CME) incidence.

**Results:**

Group 2 demonstrated significantly shorter surgical durations (110±2.54 seconds) compared to Group 1 (152±2.23 seconds, p < 0.001). The frequency of nucleus falls was considerably lower in Group 2 (p < 0.001). Postoperative BCVA and CME incidence showed no significant differences between groups. Multiple regression analysis confirmed the OZil handpiece significantly reduced surgical duration (β = -0.40, p < 0.001) without compromising safety.

**Discussion:**

The OZil handpiece’s rotational cutting mechanism offers a significant advantage in reducing surgical time while improving the followability of lens fragments, as compared to the traditional phacofragmatome. It addresses one of the key limitations of phacofragmentation by minimizing fragment displacement, where repulsive forces can complicate the procedure. Although both techniques showed similar safety profiles, the OZil handpiece’s operational efficiency makes it a promising alternative for managing posteriorly displaced lens fragments in complex cases.

**Conclusions:**

The OZil phacoemulsification handpiece significantly enhances surgical efficiency and safety in nucleus drop surgeries. Its integration into existing phacofragmatome systems can lead to major advancement in the ophthalmic surgical armamentarium, ensuring improved patient care.

## Introduction

Although phacoemulsification is the most often used technique for cataract surgery, this approach presents various complications, including lens nuclei or fragments dislodging into the vitreous cavity. Its incidence ranges from 0.1% to 1.5%, although it is a rare but serious condition [1,2]. Some complications, including persistent intraocular inflammation, cystoid macular edema (CME), retinal detachments, secondary glaucoma, corneal edema, and endophthalmitis can result from the retained lens material. All these conditions can cause permanent vision loss [3,4].

Combined with phacofragmentation, pars plana vitrectomy (PPV) has long been a standard procedure for removing displaced lens fragments [5,6]. The traditional phacofragmatome uses axial ultrasonic energy to emulsify and aspirate lens material. This back-and-forth motion can propel lens fragments away from the tip, increasing the risk of retinal damage. The use of perfluorocarbon liquids (PFCLs) in these procedures, while beneficial for mitigating retinal damage, also increases surgical costs and duration, as does the risk of complications from residual PFCLs [7].

In contrast, the OZil phacoemulsification handpiece employs torsional movement with side-to-side oscillation. This method effectively shaves lens fragments with reduced repulsive force, minimizing the risk of fragments being pushed away and reducing the likelihood of retinal damage. Torsional movement enhances the procedure’s efficiency and safety by improving the followability of lens particles and decreasing the overall surgical duration [8,9].

The literature has highlighted the potential of torsional phacoemulsification as a safer and more efficient alternative to traditional methods for managing dropped lenses. However, most supporting studies have been either retrospective or prospective case series in design, and randomized controlled trials are lacking. Our study is the first randomized controlled trial in this area, providing a robust evaluation of the efficacy and safety of the OZil phacoemulsification handpiece specifically for dropped lenses compared to the traditional phacofragmatome.

## Materials and methods

### Study Design

This prospective study was conducted in a tertiary care hospital in eastern India from 01 January 2023 to 30 June 2023. Approval from the ethical committee was obtained before the study began. The study protocol conformed to the tenets of the Declaration of Helsinki. This study was registered with the Clinical Trial Registry-India (CTRI/2023/01/049013).

### Aim of the Study

To determine the efficacy of the OZil phacoemulsification handpiece compared to traditional phacofragmatome in managing posteriorly displaced/dropped lens fragments during cataract surgery.

### Primary Objective

To estimate the effectiveness of the OZil handpiece in decreasing surgical duration and minimizing intraoperative complications, mainly the dropping of lens fragments onto the retinal surface, compared to the phacofragmatome.

### Secondary objectives

1. To estimate postoperative best corrected visual acuity (BCVA) and compare the outcomes between the two groups.
2. To estimate and compare the incidence of postsurgical CME in patients who underwent surgery.
3. To determine the safety of both procedures, the surgical site was evaluated postoperatively, focusing on noting the scleral burns or related complications.

### Study Participants

We enrolled patients who experienced nuclear dislocation during cataract surgery. Patients having retinal detachment or endophthalmitis were excluded. A thorough preoperative assessment was done for all patients. Patients with increased intraocular pressure, corneal edema, or uveitis were managed medically before the surgery. All included patients had lens nuclei grading ranging from LOCS grade II to IV [10]. Before the intervention, the duration of the Nucleus drop was also noted.

Informed consent was taken from all patients who adhered to the ethical standards and were fully aware of the nature of the study. In our study, 40 patients were enrolled and randomized using block randomization with a block size of 4. Thus, Group 1 and Group 2 consisted of 20 patients each. Group 1 patients were managed using the traditional phacofragmatome, while Group 2 patients underwent surgery using the OZil handpiece (OZil™, Alcon’s Infiniti Vision System).

### Surgical technique

**Fig. 1:**
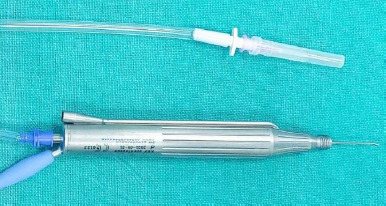
OZil phacoemulsification probe with its sleeve removed, exposing the phaco tip for phacofragmentation, alongside the infusion pipe, which is capped

All procedures were performed under local peribulbar anaesthesia using 10 ml of 2% lignocaine and bupivacaine following a thorough preanesthetic evaluation by an anaesthesiologist. This included assessing patient history, current medications, and potential risk factors to ensure safe anaesthesia administration. After administering local anaesthesia, a 25-gauge vitrectomy was performed to clear the vitreous around the dislocated lens fragments, and posterior vitreous detachment was carried out. A sclerotomy was then performed at either the superotemporal or superonasal quadrant, based on whether the surgery was on the right or left eye. The sclerotomy was made 3.5 mm posterior to the limbus using a 20-gauge microvitreoretinal (MVR) blade. Following the initial vitrectomy, additional vitrectomy was performed at the port site to ensure a clear pathway. Patients in group 1 subsequently underwent conventional phacofragmentation surgery. However, in group 2, the OZil handpiece’s infusion pipe was disconnected and securely sealed with a needle cover to prevent any leakage during insertion (**Fig. 1**). The sleeveless OZil handpiece was carefully inserted into the sclerotomy. The nuclear material was phacoemulsified using an OZil handpiece with a torsional movement technique. The operational parameters for all the cases were standardized as follows:

- The vacuum pressure was set at 250 mmHg to maintain consistent aspiration.
- The aspiration rate was set at 30 to control the flow of fluid and lens material.
- The torsional amplitude was set at 100% to maximize the effectiveness of the torsional movement.
- The phaco power was adjusted based on the density of the lens material to ensure optimal emulsification and removal.

Before concluding the procedure, the retinal periphery was meticulously examined for any iatrogenic breaks or potential damage. The sclerotomy sites were carefully closed, and any necessary additional vitrectomy was performed to ensure that no lens fragments remained in the vitreous cavity. Throughout the procedure, we avoided perfluorocarbon liquids (PFCLs). Postoperative care included monitoring for any signs of inflammation, increased intraocular pressure, or other complications. Based on individual assessments, patients were prescribed topical steroids and antiglaucoma medications as needed.

### Statistics

The sample size was calculated based on the primary outcome measure, meaning the reduction in surgical duration. For the calculation, we used a mean difference in surgical duration of 40 seconds between the two groups and a standard deviation of 10 seconds, with a power of 80% and a significance level of 0.05. Statistical analyses were done using the Statistical Package for Social Sciences (SPSS) version 29.0 software. Categorical variables were presented as percentages while continuous variables were presented as the mean ± standard deviation. We compared two groups using independent t-tests in the case of continuous variables and chi-square tests in the case of categorical variables. A p-value of < 0.05 was considered to indicate statistical significance.

## Results

### Demographics

Of the 40 patients included in the study, 22 (55%) were males, and 18 (45%) were females. There were 13 males (65%) and 7 females (35%) in Group 1 (phacofragmatome group), and 12 males (60%) and 8 females (40%) in Group 2 (OZil group). The chi-square test indicated no statistically significant difference in sex distribution between the two groups. The mean age of the participants in Group 1 was 61.44±6.4 years, while in Group 2, it was 63.56±7.5 years. An unpaired t-test revealed no statistically significant difference in age between the groups (p-value = 0.342).

### Preoperative characteristics (**Table 1**)

**Table 1:** Preoperative and intraoperative characteristics

Parameter	Group 1 (n=20)	Group 2 (n=20)	P value	Test used
**Demographics**				
Males (%)	13 (65%)	12 (60%)		
Females (%)	7 (35%)	8 (40%)		
Mean Age (years)	61.44±6.4	63.56±7.5	0.342	Unpaired t-test used
**Preoperative Characteristics**				
Mean BCVA (logMAR)	0.468±0.21	0.43±0.11	0.479	Unpaired t-test used
Cataract Grading (LOCS II-IV)				
II	6	8	0.710	Chi-square test used
III	8	8		
IV	6	4		
Time Between Lens Dislocation and Intervention	8.2±1.5 days	8.4±1.6 days	0.685	Unpaired t-test used
**Surgical Duration**	152±2.23 seconds	110±2.54 seconds	< 0.001	Unpaired t-test used
**Intraoperative Complications**				
Nucleus Fall Frequency	6 times in 4 cases, 7 times in 10 cases, 8 times in 6 cases	3 times in 5 cases, 4 times in 10 cases, 2 times in5 cases		
Nucleus Fall frequency Median (Min-Max)	7 (4-8)	3 (2-4)	< 0.001	Mann‒Whitney U test used

Note: Significant differences in surgical duration and nucleus fall frequency are observed between Group 1 and Group 2. The surgical duration was significantly longer in Group 1 compared to Group 2 (p < 0.001). Additionally, the median frequency of nucleus falls was higher in Group 1 than in Group 2 (p < 0.001), indicating a notable difference in intraoperative complications between the two groups.

The preoperative best corrected visual acuity (BCVA) on the logMAR scale, cataract grade, and time between lens dislocation and intervention were recorded. The mean preoperative BCVA was 0.468±0.21 logMAR in Group 1 and 0.43±0.11 in Group 2, with no significant difference (p = 0.479). The cataract grade (LOCS II-IV) was comparable between the groups. Group 1 included 6 patients with grade II, 8 with grade III, and 6 with grade IV, while Group 2 included 8 patients with grade II, 8 with grade III, and 4 with grade IV (p = 0.710). The mean time between lens dislocation and intervention was 8.2±1.5 days in Group 1 and 8.4±1.6 days in Group 2, which were not significantly different (p = 0.685).

### Surgical Duration

Compared with Group 1, Group 2 demonstrated superior surgical efficiency, with an average operative duration of 110±2.54 seconds, and an average operative duration of 152±2.23 seconds. This difference in surgical duration was statistically significant (p-value < 0.001).

### Intraoperative Complications (**Table 1**)

Although all patients experienced the nucleus falling onto the retinal surface at least once, the frequency of this occurrence varied significantly between the two groups. In Group 1, the nucleus fell 6 times per patient in 4 patients, 7 times per patient in 10 patients, and 8 times per patient in 6 patients. In Group 2, the nucleus fell 3 times per patient in 5 patients, 4 times per patient in 10 patients, and 2 times per patient in 5 patients. This difference in frequency was statistically significant, with a median fall frequency of 7 (4-8) in Group 1 and 3 (2-4) in Group 2 (p < 0.001).

### Postoperative BCVA (**Table 2**)

**Table 2:** Postoperative characteristics

Parameter	Group 1 (n=20)	Group 2 (n=20)	P value	Test used
**Postoperative Characteristics**				
Postoperative BCVA (logMAR)	0.112±0.054	0.118±0.051	0.719	Unpaired t-test used
PostoperativeCystoid Macular Edema (CME)				
-Incidence	5 (25%)	3 (15%)	0.429	Chi-square test used
-Mean Duration to Development	7±1.5 weeks	8±1.2 weeks	0.025	Unpaired t-test used
Surgical Site Examination	No scleral burns or related complications	No scleral burns or related complications		

The BCVA showed no significant difference between the two groups at the six-month postoperative period. The average BCVA of group 2 was 20.118±0.051 logMAR, and group 1 had a nearly similar average BCVA of 0.112±0.054 logMAR. The difference was not statistically significant (p = 0.719).

### Postoperative CME (**Table 2**)

CME was diagnosed in 5 out of 20 patients (25%) managed with phacofragmatome, while Group 2 had CME in only 3 out of 20 patients (15%). This difference was not statistically significant (p = 0.429). The mean duration of CME development in Group 1 was 7±1.5 weeks, while in Group 2, it was approximately 8±1.2 weeks (p = 0.025).

### Surgical Site Examination (Table 2)

Postoperative examination of the surgical sites revealed no scleral burns or related complications in any of the groups.

### Regression Analysis

Multiple linear regression analysis revealed that the use of the OZil handpiece was significantly associated with a reduction in surgical duration (β = -0.40, p < 0.001), even after adjusting for age, sex, preoperative BCVA, cataract grade, and time between lens dislocation and intervention. None of the covariates, except for the surgical technique, significantly affected the surgical duration.

### Logistic Regression for Postoperative Complications

Logistic regression analysis for the incidence of CME revealed no significant difference between the groups (odds ratio [OR] = 0.60, 95% confidence interval [CI] = 0.12-2.97, p = 0.52) after adjusting for age, sex, preoperative BCVA, cataract grade, and time between lens dislocation and intervention.

## Discussion

Our study on the OZil phacoemulsification handpiece demonstrates the substantial influence of technical developments in ocular surgery. Our study’s findings demonstrate the OZil handpiece’s improved safety profile and operational effectiveness when managing posteriorly displaced lens fragments. One of our noteworthy discoveries is the significant decrease in surgical time that we could accomplish using the OZil handpiece in contrast to the conventional phacofragmatome. Specifically, the mean surgical duration for the OZil handpiece group was significantly shorter (110±2.54 seconds) than that for the Phacofragmatome group (152±2.23 seconds, p < 0.001).

One of the main advantages of the OZil handpiece is its unique cutting technique. OZil uses a rotational cutting approach in contrast to the conventional phacoemulsification procedure, which depends on a back-and-forth motion [11]. This method prevents chatter by successfully trimming the lens fragments in a shaving-like manner instead of displacing them. As a result, the fragments are less likely to be pushed away, which is a key problem with the phacofragmatomes. Instead, they are likely to stay close to the instrument’s tip. Additionally, this feature improves the lens material’s followability.

**Fig. 2:**
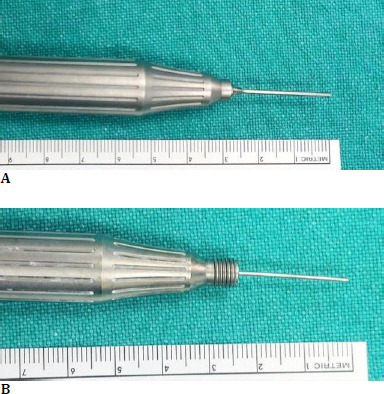
**A** Illustration of the conventional fragmatome with a phaco tip measuring 22.5 mm, in contrast to the OZil tip’s length of 20 mm, as depicted in **B**

The conventional fragmatome tip is longer (22.5 mm) (**Fig. 2 A**) than the OZil tip (20 mm), (**Fig. 2 B**) [12]. However, we hypothesize that patients with high axial myopia may find it more difficult to reach the retinal surface. One advantage of the OZil handpiece is that it does not tend to clog the tip. We observed a more noticeable “milking” phenomenon with traditional handpieces, which was observed much with OZil. Overall, the OZil handpiece shows better efficiency and safety in handling lost lens nuclei when compared to typical phacofragmentation approaches.

Multiple linear regression analysis revealed that the OZil handpiece was significantly associated with a reduction in surgical duration, which showed that the OZil handpiece independently contributed to reducing surgical duration, underscoring its operational efficiency. The lack of significant effects from other covariates further emphasizes that the choice of surgical technique plays a crucial role in determining the procedure’s efficiency. Logistic regression analysis for the incidence of CME revealed no significant difference between the groups. This suggests that the choice of surgical technique (OZil vs. Phacofragmatome) does not significantly influence the likelihood of developing CME postoperatively.

This result supports the comparable safety profiles of the two methods, indicating that, while the OZil handpiece improves operational efficiency, it does not compromise patient safety.

During our search on PubMed, Embase, and Scopus for studies, we found very few publications comparing the OZil handpiece to the phacofragmatome. One of these studies was a prospective analysis of 15 eyes using the OZil handpiece. However, unlike our research, this study lacked a comparison group. The authors found no retinal injury or scleral thermal burns, indicating that the OZil torsional handpiece is a secure and reliable alternative for conventional longitudinal phacofragmentation [8]. Another retrospective study by Garg et al., on a cohort of 12 patients, showed similar findings, highlighting the removal of the dropped lens using an OZil handpiece [9]. Chiang et al. also reported a retrospective case series of 34 patients [13]. They reported excellent followability of the OZil handpiece, similar to our study. Notably, their mean total operating time (without vitrectomy) for removing residual lens material was 111 seconds, which is faster than our study’s results. Moreover, our findings’ congruence with earlier research lends additional validity to our findings. Being the only study combining a comparison group and a prospective method, ours stands out in the literature.

Our study has a few limitations. The fact that it was conducted at a single tertiary care hospital may have limited the findings’ wider relevance and generalizability to various healthcare settings. Furthermore, the accessibility and availability of the OZil phacoemulsification handpiece in different healthcare settings were not examined in the study, which could have influenced its usefulness in a range of ophthalmic surgical settings. The lack of a thorough cost-benefit analysis contrasting the usage of the OZil handpiece with that of the traditional phacofragmatome is another noteworthy shortcoming, especially considering the need for a full vitrectomy system.

The strengths of our study lie in its prospective design as we could collect data and observations in real time by conducting it prospectively, which increased the reliability and validity of our conclusions. An important feature of this study is the comparison group addition, which compares the OZil handpiece with the traditional phacofragmatome. With this comparative method, we better understand how well the OZil handpiece functions in surgical situations. Strict data collection and analysis methods reinforce the validity of our findings, providing strong evidence for the efficacy and promise of the OZil handpiece in current ophthalmic procedures.

## Conclusion

In conclusion, our research demonstrated that the OZil phacoemulsification handpiece represents a valuable innovation for nucleus drop surgeries due to its enhanced efficiency and safety. Notably, this study underscores the potential for the side-to-side motion mechanism of the OZil handpiece to be adapted into phacofragmatome handpieces. Such integration could lead to significant advancements in surgical instruments for ophthalmologists. Additionally, the OZil handpiece offers a dependable alternative in cases where a phacofragmatome is unavailable or experiences malfunction during a procedure. Embracing and modifying this technology will not only leverage the benefits of both devices but also promote further advancements in surgery and enhance patient outcomes.

### What was known before

Managing dropped lens nuclei during cataract surgery poses significant risks. Traditional methods involve pars plana vitrectomy with phacofragmentation, which can increase retinal damage risk due to repulsive movements.

### What this study adds

The OZil handpiece significantly reduces surgical duration intraoperative complications through its rotational movements during nucleus drop surgery, compared to the traditional phacofragmatome.
